# The imaging-quiescent phenotype in thrombolysed acute ischemic stroke: a diffusion-weighted MRI–based deconstruction

**DOI:** 10.3389/fnins.2026.1925929

**Published:** 2026-08-26

**Authors:** Songqi Hou, Rui Wang, Xiaobin Chen, Zetuo Wang, Huaiyi Li, Yanwei Wang, Gen Yan

**Affiliations:** 1 Department of Radiology, The Second Affiliated Hospital of Xiamen Medical College, Xiamen, China; 2 Department of Neurology, The Second Affiliated Hospital of Xiamen Medical College, Xiamen, China

**Keywords:** acute ischemic stroke, CT perfusion, diffusion-weighted MRI, intravenous thrombolysis, phenotype classification, stroke mimics

## Abstract

**Background:**

Standard CT perfusion mismatch and hypoperfusion intensity ratio formulas are undefined when ischemic core or Tmax>6 s equals zero. Such “imaging-quiescent” patients remain uncharacterized. We quantified their prevalence and used DWI to separate CTP-undetected ischemia from DWI-negative presentations.

**Methods:**

We conducted a single-center retrospective cohort study of 188 consecutive patients receiving intravenous thrombolysis between January 2023 and October 2025. Patients were classified into four CTP phenotypes based on the presence of rCBF<30% core and Tmax>6 s hypoperfusion: A (classic mismatch), B (core without hypoperfusion), C (pure penumbra), and D (imaging-quiescent, both volumes = 0 mL). Group D was further subdivided by post-thrombolysis DWI into D1 (DWI-positive), D2 (DWI-negative), and D3 (DWI unavailable). The primary outcome was 90-day modified Rankin Scale 0–2. Analyses included Kruskal–Wallis tests, multivariable logistic regression, two one-sided tests, and false discovery rate adjustment.

**Results:**

Of 188 thrombolysed patients, 168 had classifiable CTP phenotypes and 161 had 90-day mRS data. Group D was the largest phenotype (*n* = 69, 41.1%), with a favorable outcome rate of 84.6%, comparable to Group C (83.9%) and higher than Group A (55.9%). Among 62 Group D patients with DWI, 42 (60.9%) were DWI-positive (D1) and 20 (29.0%) were DWI-negative (D2). D1 patients had higher NIHSS (median 4 vs. 2), more posterior circulation involvement (38.1% vs. 5.0%), and lower favorable outcome rates (80.5% vs. 94.1%); 65% of D2 patients achieved mRS = 0. Group A had 51.4% large-vessel occlusion versus 4.4% in Group D. In multivariable regression (EPV = 17, AUC = 0.823), NIHSS was the strongest predictor of favorable outcome (OR 0.83 per point, 95% CI 0.75–0.91; *p* < 0.001).

**Conclusion:**

The imaging-quiescent phenotype is not a single entity but a composite of three operationally defined subgroups (D3 reflecting absent MRI data rather than a distinct biological entity), with approximately 30% being DWI-negative, a population enriched for stroke mimics or transient ischemic events. DWI reclassified nearly one-third of CTP-quiescent patients and may improve diagnostic precision. These findings are hypothesis-generating and suggest that post-thrombolysis DWI may improve diagnostic characterization of CTP-quiescent patients; prospective study is required before any change to pre-thrombolysis workflow can be recommended.

## Introduction

Computed tomography perfusion (CTP) imaging has reshaped acute ischemic stroke (AIS) triage since DEFUSE-3 and EXTEND-IA established that quantitative mismatch profiles—defined by ischemic core (rCBF<30%) volumes and hypoperfusion (Tmax>6 s) volumes—predict reperfusion benefit in late-window large-vessel occlusion (Albers et al., 2018; Campbell et al., 2015). Subsequent trials [DAWN (Nogueira et al., 2018), EXTEND (Ma et al., 2019), WAKE-UP (Thomalla et al., 2018), TIMELESS (Albers et al., 2024), TRACE-III (Xiong et al., 2024), CHABLIS-T II (Cheng et al., 2025)] have extended perfusion-imaging selection to intravenous thrombolysis in the extended time window, and the 2026 AHA/ASA guideline endorses extended-window thrombolysis based on advanced imaging (Prabhakaran et al., 2026).

**Figure 1:**
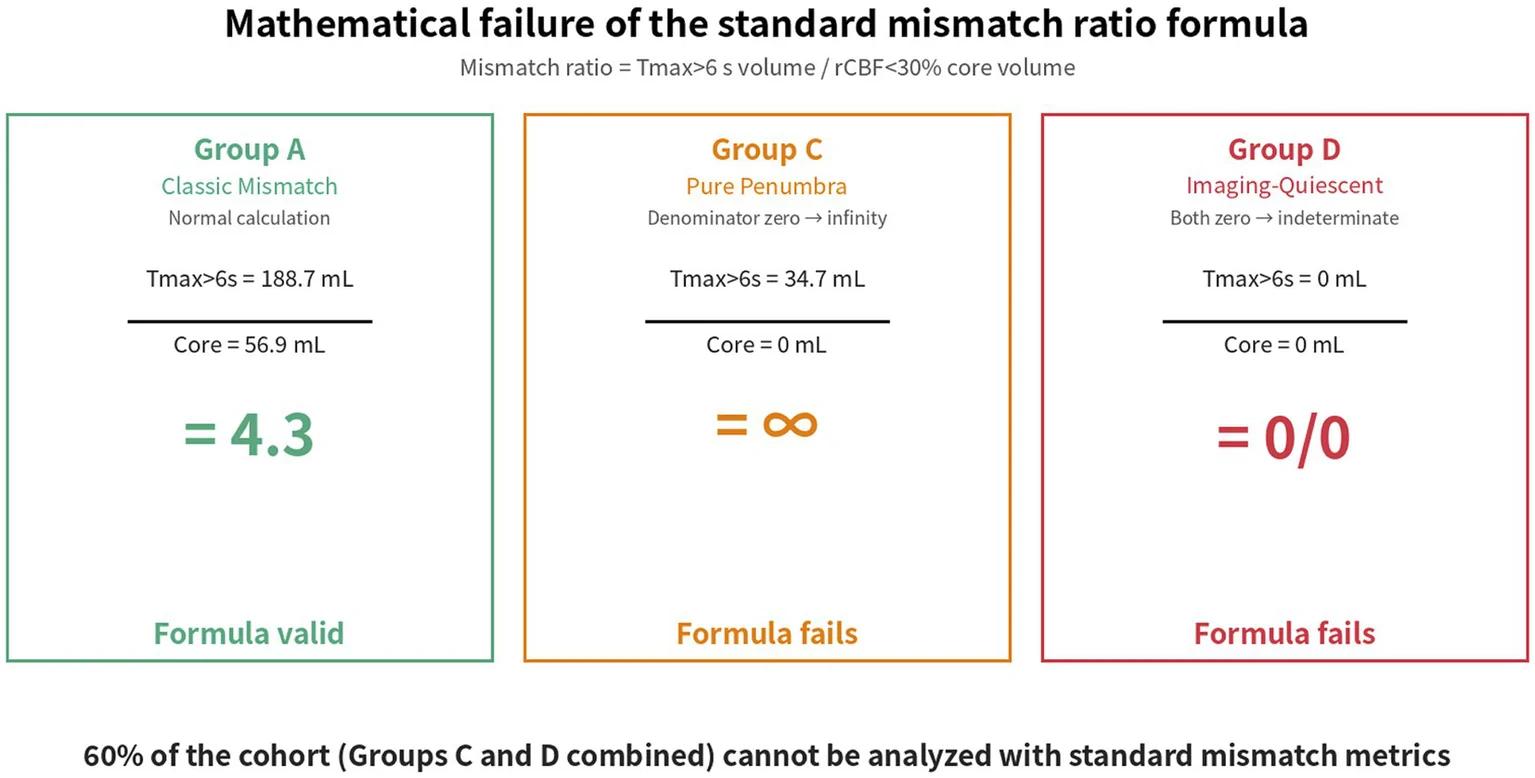
Visual evidence for the mathematical failure of the standard mismatch ratio formula. In Group A the formula yields a clinically meaningful value (4.3); in Group C the zero denominator yields infinity (rendered as “infinity” by the clinical software); in Group D the indeterminate form 0/0 yields a software label of “undefined.” This failure mode is not a statistical inconvenience but a routine occurrence in real CTP output, observable in 60% of our cohort. CTP indicates CT perfusion; rCBF, relative cerebral blood flow; Tmax, time to maximum.

These pivotal trials enrolled patients with substantial neurological deficits and large-vessel occlusion. Real-world AIS populations are clinically broader, with mild stroke (NIHSS ≤5) accounting for nearly half of all ischemic stroke presentations (Khatri et al., 2018). Within this broader spectrum lies a population for whom standard CTP-derived metrics cannot generate a numerical descriptor at all: patients with both ischemic core and Tmax>6 s volumes equal to zero. Because the mismatch ratio (penumbra/core) and hypoperfusion intensity ratio (HIR; Tmax>10 s/Tmax>6 s) (Olivot et al., 2009, 2014) both rely on these quantities as denominators, such “imaging-quiescent” patients are mathematically excluded from analyses derived from standard CTP workflows. Importantly, these two ratios fail under different conditions: the mismatch ratio is undefined whenever core = 0, whereas HIR remains computable as long as Tmax>6 s > 0 and becomes undefined only when Tmax>6 s = 0. An undefined ratio therefore reflects a mathematical limitation of a derived metric rather than a failure of CTP imaging itself. As demonstrated in Figure 1, this mathematical failure is not a theoretical concern but a practical reality readily observable in routine clinical CTP output: software displays “∞” (infinity) when the core denominator is zero, and “undefined” when both volumes are zero.

The individual components of this problem are not themselves new observations. CTP is known to yield false-negative results, and several groups have quantified this directly. In a large consecutive series using automated RAPID post-processing, 57% of patients with a negative CTP nevertheless received a final clinical diagnosis of stroke, with false negatives attributable predominantly to lacunar supratentorial infarction and vertebrobasilar stroke, and a further subset located outside the CTP field of view (Cviková et al., 2024). Dedicated studies of lacunar stroke have shown that CTP has high specificity but low sensitivity for lacunar infarction, performing particularly poorly in the basal ganglia and thalamus (Benson et al., 2016). Comparative software work has further demonstrated that the ability of automated packages to exclude small infarcts varies substantially between vendors and threshold settings, so that a reported core volume of zero is in part a property of the processing pipeline rather than of the patient (Thormann et al., 2025). Conversely, DWI itself is imperfect: a meta-analysis of 3,236 patients found a pooled prevalence of DWI-negative ischemic stroke of 6.8%, and DWI-negative stroke was five times more likely in posterior-circulation ischemia (Edlow et al., 2017). Discordance between CTP and DWI in thrombolysed patients has also been reported, including in cohorts examining perfusion deficits among DWI-negative and DWI-positive patients (Zhu et al., 2023).

What has not been established is how these separate observations combine within the specific population defined by a mathematically undefined CTP profile. Prior work has generally treated CTP negativity as a binary diagnostic property, asking how often a negative CTP misses a stroke. It has not characterized patients in whom both perfusion volumes are simultaneously zero as a distinct, prospectively identifiable imaging phenotype, quantified how frequently that phenotype occurs within an unselected thrombolysed cohort, or partitioned it by DWI status to separate CTP-undetected ischemia from non-ischemic presentations. The novelty of the present work is therefore not the discovery that CTP can be negative in true stroke, which is established, but the systematic characterization of a specific and common CTP output state—one in which standard derived metrics cannot be computed at all—together with an empirical estimate of what that state contains.

The biological plausibility of clinically symptomatic stroke with completely normal CTP requires careful consideration. Several mechanisms have been proposed: sub-CTP-resolution lesions (small lacunar or cortical infarcts beneath the spatial sensitivity of perfusion processing); spontaneously reperfused embolic events leaving deficit but no perfusion abnormality; posterior circulation strokes in territories where CTP coverage and sensitivity are limited; and stroke mimics (e.g., complicated migraine, post-ictal paresis, functional disorders, hypoglycemia) that present with deficits but lack ischemic pathophysiology. Distinguishing among these mechanisms in a single CTP-only cohort has been infeasible, leaving the imaging-quiescent phenotype as a speculative entity. Diffusion-weighted MRI (DWI), with its substantially higher sensitivity for small acute infarcts—a highly sensitive but not absolute marker that cannot entirely exclude ischemia (Schellinger et al., 2010), offers a path to empirical deconstruction.

In this study, we leveraged a real-world thrombolysis cohort with paired CTP and post-thrombolysis DWI data to (1) quantify the relative frequencies of four CTP-defined phenotypes; (2) compare clinical characteristics, treatment intensity, and 90-day outcomes across phenotypes; and (3) empirically deconstruct the imaging-quiescent phenotype by DWI status, separating CTP-undetected ischemia from DWI-negative presentations enriched for stroke mimics and transient events.

## Methods

### Data availability statement

De-identified individual-patient data and analysis code that support the findings of this study are available from the corresponding author upon reasonable request and following institutional review.

### Study design and patients

We conducted a single-center, retrospective cohort study of consecutive AIS patients receiving intravenous thrombolysis between January 1, 2023 and October 31, 2025 at our comprehensive stroke center. All patients underwent baseline multimodal CT including non-contrast CT, CT angiography, and CTP before thrombolysis. Inclusion criteria were age ≥18 years, ischemic stroke diagnosis, intravenous alteplase (0.9 mg/kg) or tenecteplase (0.25 mg/kg) within current guideline-recommended time windows, availability of CTP-derived volumetric data, and availability of follow-up clinical assessment. Patients with missing core volume, hypoperfusion volume, NIHSS, or follow-up data were excluded from corresponding analyses on a per-analysis basis. This study was conducted in accordance with the Declaration of Helsinki and approved by the Ethics Committee of The Second Affiliated Hospital of Xiamen Medical College (No. 2025020). Informed consent was waived due to the retrospective design and de-identified analysis.

### Imaging acquisition and phenotype definition

**Figure 2:**
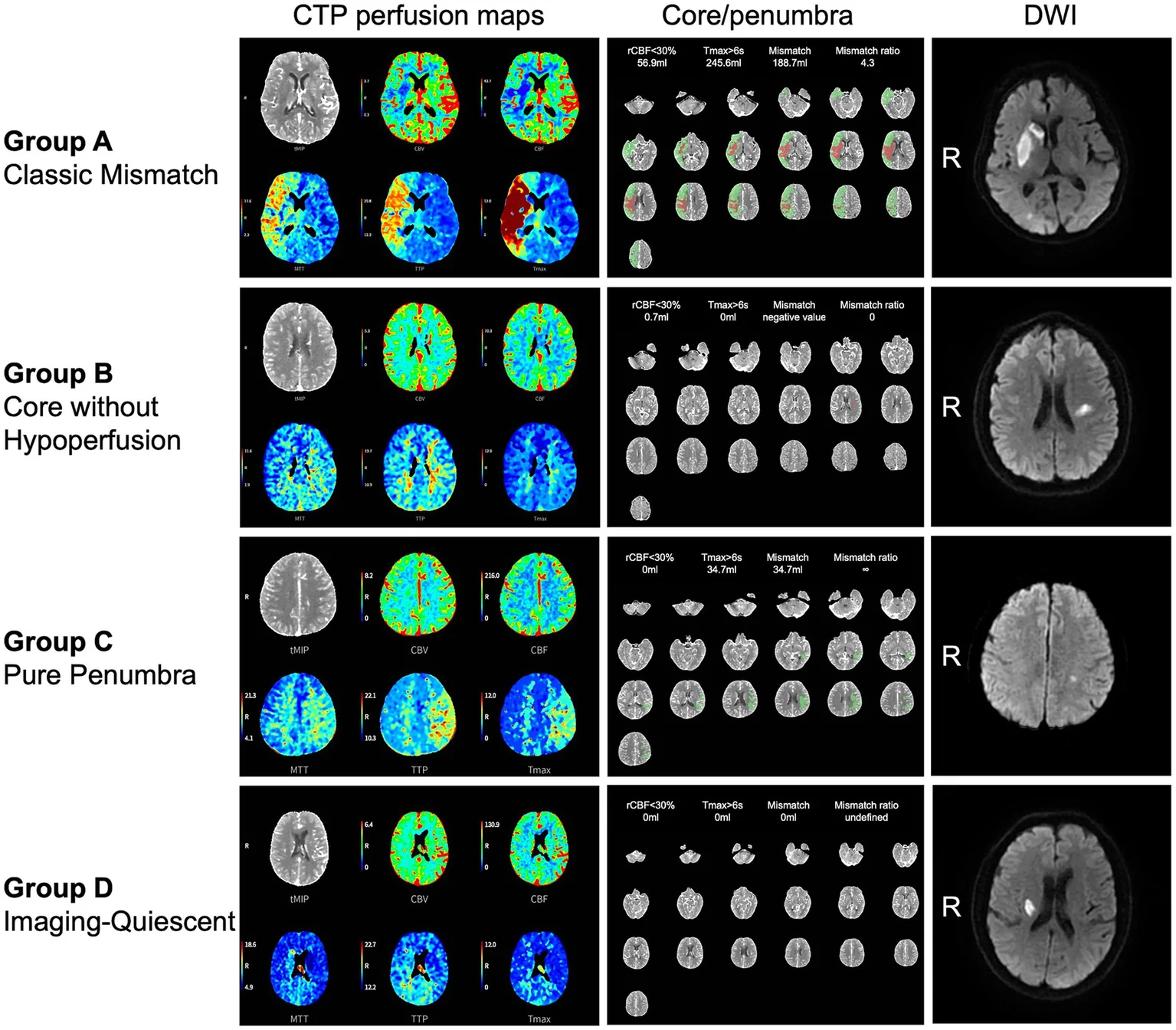
Representative CTP perfusion maps, automated core/penumbra segmentation, and DWI across the four CTP-defined phenotypes. The software-displayed mismatch ratio (top-right of each panel) reveals the mathematical failure of the standard formula in 60% of phenotypes: Group C displays “infinity” because the core denominator is zero, and Group D displays “undefined” because both quantities are zero. Group A: Core 56.9 mL, Tmax>6 s 245.6 mL, mismatch ratio 4.3. CTP shows right frontotemporoparietal hypoperfusion with a right basal ganglia core; DWI confirms a right basal ganglia infarct; Group B: Core 0.7 mL, Tmax>6 s 0 mL. CTP shows a small left corona radiata core without surrounding hypoperfusion; DWI confirms a left corona radiata infarct; Group C: Core 0 mL, Tmax>6 s 34.7 mL. CTP shows left frontoparietal hypoperfusion without a measurable core; DWI demonstrates a left frontal lobe infarct; Group D: Core 0 mL, Tmax>6 s 0 mL. CTP shows no detectable core or hypoperfusion, yet DWI reveals a right corona radiata infarct—illustrating CTP-undetected ischemia within the imaging-quiescent phenotype.

Multimodal CT was performed on a 128-slice scanner (SOMATOM Definition AS; Siemens Healthcare, Forchheim, Germany). Non-contrast CT used 100 kVp with CARE Dose4D automatic tube-current modulation and 5 mm slice thickness. CT angiography used 100 kVp, CARE Dose4D, pitch 1.4, rotation time 0.33 s, and 64 × 0.6 mm collimation, with weight-adjusted iomeprol 400 (Bracco) injected at 4.0–5.0 mL/s followed by a 40 mL saline flush, and bolus-triggered acquisition beginning 2 s after the ascending-aorta region of interest reached 150 HU. CTP used a periodic 4D spiral (toggling-table) acquisition (80 kVp, 165 mAs, rotation time 0.3 s; 96 mm z-axis coverage; 34 dynamic phases at 1.5 s temporal resolution over 53 s with a 5 s delay), with 40 mL iomeprol 400 and 40 mL saline injected at 5 mL/s. Source images were post-processed with an automated AI-assisted platform (CTPDoc, Shukun Technology, Beijing, China) using a delay-insensitive deconvolution algorithm to generate ASPECTS and CTP volumetric maps. Consistent with the manuscript’s DEFUSE-3/EXTEND-IA–based framework, ischemic core was defined as rCBF<30% relative to the contralateral hemisphere and critical hypoperfusion as Tmax>6 s; these two volumes formed the basis of phenotype classification. The 0 mL boundary was used operationally to identify patients in whom standard mismatch/HIR ratios become mathematically undefined; we acknowledge that near-zero volumes may be affected by software settings, segmentation variability, and measurement noise, and interpret the classification accordingly. This threshold definition is consistent with DEFUSE-3 (Albers et al., 2018) and EXTEND-IA (Campbell et al., 2015) criteria. Patients were classified by joint presence of these two volumes into four phenotypes: Group A (classic mismatch, core >0 mL and Tmax>6 s > 0 mL); Group B (core without hypoperfusion, core >0 mL and Tmax>6 s = 0 mL, consistent with spontaneous reperfusion); Group C (pure penumbra, core = 0 mL and Tmax>6 s > 0 mL); and Group D (imaging-quiescent, both volumes = 0 mL). Representative CTP image sets from each phenotype were reviewed to confirm the visual basis of the classification (Figure 2).

### Diffusion-weighted MRI and Group D deconstruction

Post-thrombolysis brain MRI including DWI sequences was performed when clinically feasible (typically within 24–72 h). DWI lesion presence was independently assessed by two radiologists, both blinded to CTP findings and to the clinical diagnosis, and categorized as DWI-positive (any acute restricted-diffusion lesion attributable to current ischemic event), DWI-negative (no acute restricted-diffusion lesion), or DWI not performed. Disagreements were resolved by adjudication from a third senior radiologist. Inter-reader agreement was not formally quantified, which we acknowledge as a limitation. Group D patients were further subclassified as follows:

- *D1 (DWI-positive imaging-quiescent)*: CTP showed no measurable core or hypoperfusion, but DWI confirmed an acute ischemic lesion. This category captures CTP-undetected true ischemia, including sub-resolution lesions and posterior circulation events.
- *D2 (DWI-negative imaging-quiescent)*: Neither CTP nor DWI demonstrated an acute ischemic substrate. This DWI-negative category is enriched for stroke mimics and transient ischemic events with full resolution but, in the absence of independent clinical adjudication and follow-up imaging, cannot be equated with confirmed stroke mimics.
- *D3 (DWI not performed)*: Group D patients without follow-up DWI; analyzed separately due to indeterminate underlying mechanism.

Posterior circulation involvement was identified independently from the MR descriptive report by predefined anatomical keywords (pontine, medullary, cerebellar, basilar, vertebral, posterior circulation, occipital, thalamic). Large-vessel occlusion (LVO) status was extracted from CT angiography reports.

### Outcomes

The primary outcome was a favorable functional outcome at 90 days, defined as modified Rankin Scale (mRS) 0–2. Secondary outcomes were excellent outcome (mRS 0–1), poor outcome (mRS ≥ 3), severe disability or death (mRS ≥ 5), and use of mechanical thrombectomy as bridging therapy. 90-day mRS assessments were obtained from outpatient follow-up or structured telephone interviews.

### Statistical analysis

Continuous variables are reported as median [interquartile range, IQR] and categorical variables as n (%). Across-group comparisons used the Kruskal–Wallis test (continuous) or *χ*^2^/Fisher exact test (categorical). For the pre-specified pairwise comparison of Groups C and D, we applied two one-sided tests (TOST) for equivalence in the 90-day favorable-outcome (mRS 0–2) rate. The ±10% margin was adopted as the primary equivalence bound, consistent with the absolute risk-difference margins (5–10%) commonly pre-specified in acute-stroke functional-outcome non-inferiority trials (Horvath et al., 2023). The wider ±15% margin was reported as a sensitivity analysis, acknowledging the modest subgroup sizes; presenting both conveys the robustness of the equivalence inference to the choice of margin. Predictors of favorable 90-day outcome were modeled with multivariable logistic regression adjusting for NIHSS, age, sex, ASPECTS, and phenotype (Group A as reference). Model discrimination was quantified by area under the receiver operating characteristic curve (AUC). To evaluate potential selection bias arising from non-uniform MRI acquisition, baseline characteristics, procedural metrics, and 90-day outcome were compared between Group D patients who did and did not undergo post-thrombolysis MRI (Mann–Whitney U for continuous, Fisher exact for categorical variables; Supplementary Table S1). Multicollinearity among model covariates was assessed using variance inflation factors (VIF; Supplementary Table S2). To assess robustness to multiple testing, we applied Benjamini–Hochberg false discovery rate (FDR) adjustment to all primary across-phenotype *p* values. All tests were two-sided with *α* = 0.05. Analyses used Python 3.11 with statsmodels v0.14 and scipy v1.11.

## Results

### Visual anchoring of the four phenotypes

Representative CTP imaging from each of the four phenotype groups is shown in Figure 2. The Classic Mismatch case (Group A; case ZQ) demonstrates the canonical reperfusion-eligible profile: a 56.9 mL ischemic core, a 245.6 mL Tmax>6 s hypoperfusion, a mismatch volume of 188.7 mL, and a mismatch ratio of 4.3—well within the DEFUSE-3 / EXTEND-IA selection criteria. The Core without Hypoperfusion case (Group B; case WC) shows a tiny 0.7 mL ischemic core without any measurable hypoperfusion deficit, consistent with a small completed infarct with spontaneous reperfusion; the software returns a mismatch volume labeled “negative value” and a mismatch ratio of 0.0. The Pure Penumbra case (Group C; case HH) shows zero core and 34.7 mL of hypoperfusion, with the software displaying a mismatch ratio of “infinity” — the literal numerical failure of the formula when the denominator is zero. The Imaging-Quiescent case (Group D; case LJ) shows both core and Tmax>6 s volumes of 0 mL on the standard rCBF<30% threshold, with the software displaying mismatch ratio “undefined.”

This visual evidence directly motivates the analytical framework of this study. As shown in Figure 1, the standard mismatch ratio (Tmax>6 s/core) is computable and clinically meaningful only in Group A; in Groups B, C, and D—which collectively comprise approximately 64% of the cohort—the formula produces values that are mathematically invalid or biologically uninterpretable.

### Cohort and phenotype distribution

Of 188 consecutively thrombolysed patients between January 2023 and October 2025, 168 had complete CTP volumetric data permitting phenotype classification. 90-day mRS was available for 161 patients (95.8% of classifiable cohort), and DWI status was determinable for 175 patients (93.1% of total cohort). The cohort had a median age of 61 years (IQR 51–71), 70.2% male, median admission NIHSS 6 (IQR 3–10), and overall 90-day favorable outcome rate of 74.5%.

**Figure 3:**
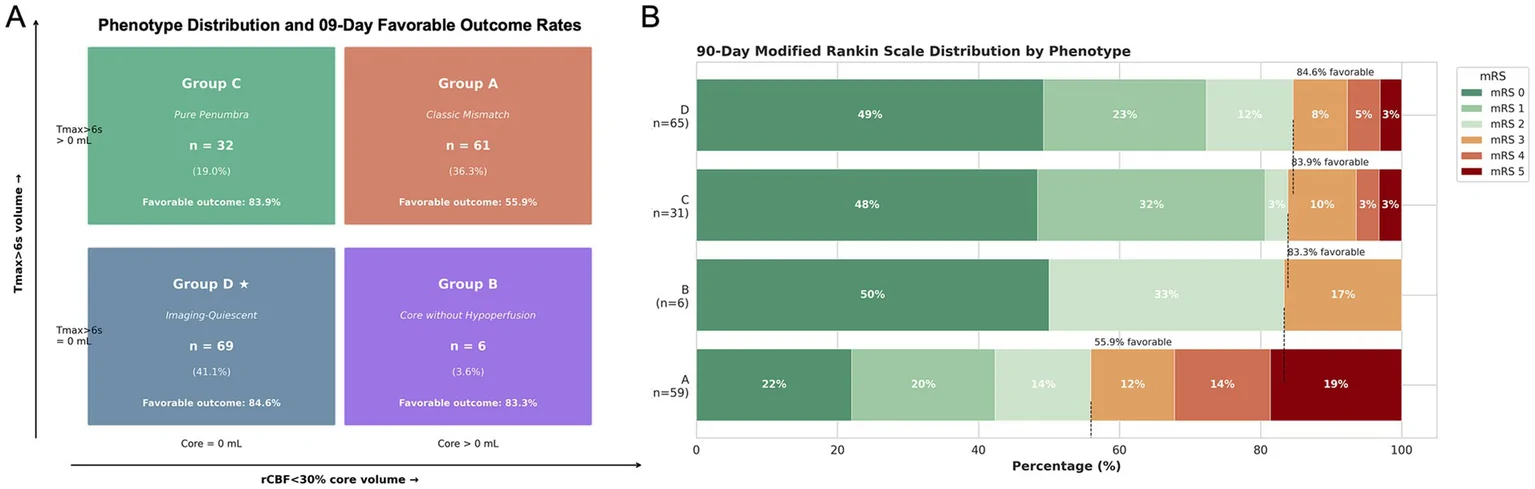
**(A)** CTP-defined phenotype matrix in 168 classifiable patients. Group D (imaging-quiescent, both rCBF <30% core and Tmax>6 s volumes = 0 mL) constituted the largest single phenotype (41.1%, *n* = 69), exceeding the classic mismatch phenotype (Group A, 36.3%, *n* = 61). **(B)** Distribution of 90-day modified Rankin Scale (mRS) scores by phenotype. The vertical dashed line within each bar indicates the boundary between favorable (mRS 0–2) and unfavorable (mRS ≥ 3) outcomes. Group A (classic mismatch) carries the highest burden of severe disability, while Groups B, C, and D show similarly high favorable outcome rates (83–86%). CTP indicates CT perfusion; rCBF, relative cerebral blood flow; Tmax, time to maximum.

The four-phenotype distribution is depicted in Figure 3A. The imaging-quiescent phenotype (Group D) was the single largest, comprising 69 patients (41.1% of the classifiable cohort), followed by classic mismatch (Group A; *n* = 61, 36.3%), pure penumbra (Group C; *n* = 32, 19.0%), and core without hypoperfusion (Group B; *n* = 6, 3.6%).

### Baseline characteristics

Baseline characteristics by phenotype are presented in Table 1. Admission NIHSS differed markedly across phenotypes (median 10 in Group A vs. 4 in Group D, raw *p* < 0.001; FDR-adjusted *p* < 0.01), as did ASPECTS and mechanical thrombectomy utilization. Large-vessel occlusion status provided important external validation of the phenotype scheme: among the 106 patients with LVO determination, Group A had a 51.4% LVO rate compared with 20.0% in Group C and only 4.4% in Group D (*p* < 0.001).

**Table 1:** Baseline characteristics by CTP-defined phenotype.

Variable	Group A (*n* = 61)	Group B (*n* = 6)	Group C (*n* = 32)	Group D (*n* = 69)	*P* (raw)	*P* (BH)
Age, years	62 [54–71]	54 [49–58]	61 [54–68]	60 [49–69]	0.45	0.72
Male sex, *n* (%)	44 (72.1)	5 (83.3)	23 (71.9)	46 (66.7)	0.80	0.94
Hypertension, *n* (%)	51 (83.6)	5 (83.3)	27 (84.4)	58 (84.1)	1.00	1.00
Diabetes, *n* (%)	18 (29.5)	1 (16.7)	11 (34.4)	20 (29.0)	0.73	0.96
Atrial fibrillation, *n* (%)	13 (21.3)	1 (16.7)	7 (21.9)	4 (5.8)	0.04	0.08
Admission NIHSS	10 [6–14]	4 [2–6]	5 [3–8]	4 [2–6]	<0.001	<0.01
ASPECTS	9 [8–10]	10 [10–10]	10 [9–10]	10 [10–10]	<0.001	<0.01
Core volume (rCBF<30%), mL	21.4 [4.5–48.4]	1.6 [0.6–3.4]	0 (by def.)	0 (by def.)	<0.001	<0.01
Tmax>6 s volume, mL	132.5 [50.6–272.7]	0 (by def.)	72.4 [23.7–142.6]	0 (by def.)	<0.001	<0.01
Large-vessel occlusion, *n*/*N* (%)	19/37 (51.4)	0/4 (0)	4/20 (20.0)	2/45 (4.4)	<0.001	<0.01
Onset-to-treatment time, min	140 [88–195]	165 [140–190]	175 [125–249]	162 [100–230]	0.51	0.77
Mechanical thrombectomy, *n* (%)	28 (45.9)	0 (0.0)	6 (18.8)	3 (4.3)	<0.001	<0.01

Continuous variables: median [interquartile range]; categorical variables: *n* (%). *p* values from Kruskal–Wallis (continuous) or *χ*^2^/Fisher exact (categorical) tests. BH = Benjamini–Hochberg false discovery rate–adjusted p value. Large-vessel occlusion denominators reflect only patients with available CT angiography assessment. ASPECTS indicates Alberta Stroke Program Early CT Score; CTP, CT perfusion; NIHSS, National Institutes of Health Stroke Scale; rCBF, relative cerebral blood flow; Tmax, time to maximum.

### 90-day functional outcomes by phenotype

The 90-day favorable outcome rates differed markedly across phenotypes (Figure 3B; Table 2). Group A had the worst prognosis (55.9% favorable, 18.6% severe disability/death), while Groups B (83.3%), C (83.9%), and D (84.6%) all achieved favorable outcomes in over 83% of cases. A formal TOST equivalence test between Groups C and D yielded *p* = 0.12 at the ±10% margin and *p* = 0.04 at the ±15% margin (90% CI of difference, −13.9 to +12.4%), indicating that the data are compatible with equivalence within a moderately wide margin but cannot confirm equivalence at the more stringent ±10% criterion typically applied to functional outcome trials.

**Table 2:** Ninety-day functional outcomes and treatment intensity by phenotype.

Outcome/Treatment	Group A (*n* = 59*)	Group B (*n* = 6)	Group C (*n* = 31*)	Group D (*n* = 65*)
Excellent outcome (mRS 0–1), *n* (%)	26 (44.1)	5 (83.3)	25 (80.6)	48 (73.8)
Favorable outcome (mRS 0–2), *n* (%)	33 (55.9)	5 (83.3)	26 (83.9)	55 (84.6)
Poor outcome (mRS ≥ 3), *n* (%)	26 (44.1)	1 (16.7)	5 (16.1)	10 (15.4)
Severe disability/death (mRS ≥ 5), *n* (%)	11 (18.6)	0 (0)	1 (3.2)	2 (3.1)
Mechanical thrombectomy, *n* (%)	28 (45.9)	0 (0)	6 (18.8)	3 (4.3)

*Numerators reflect patients with available 90-day mRS within each phenotype. mRS indicates modified Rankin Scale.

### Empirical deconstruction of the imaging-quiescent phenotype by DWI status

**Figure 4:**
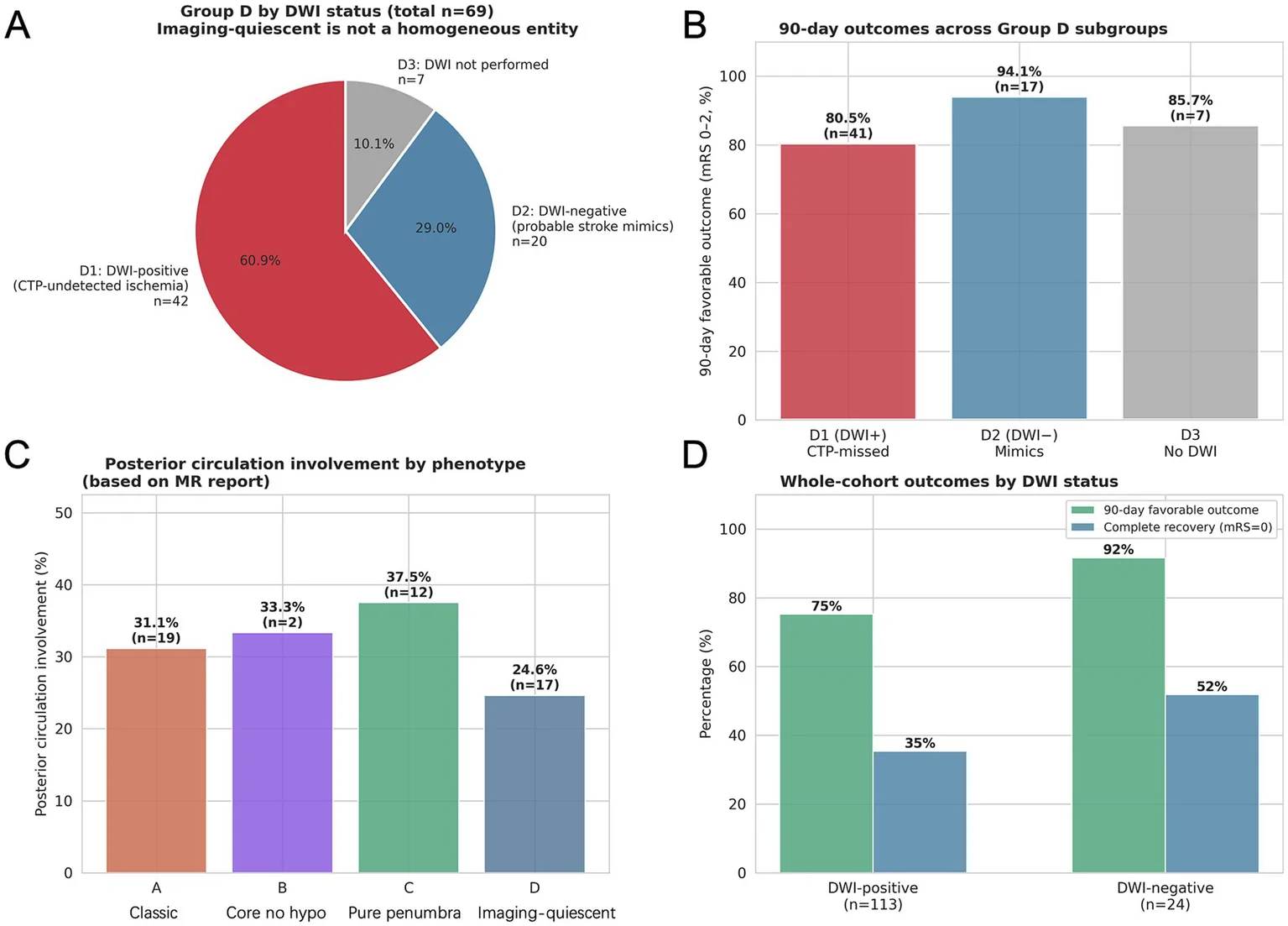
Empirical deconstruction of the imaging-quiescent (Group D) phenotype by DWI status. **(A)** Distribution by DWI status: 60.9% DWI-positive (D1), 29.0% DWI-negative (D2), 10.1% DWI not performed (D3). **(B)** 90-day favorable outcome rates across the three Group D subgroups. **(C)** Posterior circulation involvement across the four CTP phenotypes—Notably elevated in Group D, particularly the DWI-positive subgroup. **(D)** Whole-cohort comparison of 90-day favorable outcome and complete recovery (mRS = 0) rates between DWI-positive and DWI-negative patients. DWI indicates diffusion-weighted imaging; mRS, modified Rankin Scale.

Among the 69 Group D patients, 62 had determinable post-thrombolysis DWI status (89.9% ascertainment). The imaging-quiescent phenotype was internally heterogeneous and resolved into three operationally defined subgroups (D1 and D2 differing biologically by DWI status, whereas D3 reflects absence of MRI data rather than a distinct biological entity) (Figure 4; Table 3):

- *D1—DWI-positive imaging-quiescent (n = 42, 60.9% of Group D)*: CTP showed no measurable core or hypoperfusion, yet DWI confirmed an acute ischemic lesion. These patients had median NIHSS 4 (IQR 2–7), median ASPECTS 10, and a 90-day favorable outcome rate of 80.5%. Sixteen of 42 (38.1%) had posterior circulation involvement on MR description — comprising 10 patients with unequivocal brainstem, cerebellar, or occipital lesions and 6 with thalamic lesions classified as posterior-circulation on the basis of predominant perforator supply — consistent with the well-recognized limitation of standard anterior-circulation–optimized CTP protocols in detecting brainstem, cerebellar, and thalamic lesions.
- *D2—DWI-negative imaging-quiescent (n = 20, 29.0% of Group D)*: Neither CTP nor DWI demonstrated an acute ischemic substrate. These patients had the lowest NIHSS (median 2, IQR 1–4) and the highest 90-day favorable outcome rate (94.1%); 13 of 20 (65.0%) reached mRS = 0 (complete recovery). Only 1 of 20 (5.0%) had posterior circulation involvement on MR description, and none had documented LVO. This profile is compatible with stroke mimics or transient ischemic events with full clinical and radiologic resolution, although DWI negativity alone cannot establish either diagnosis.
- *D3—DWI not performed (n = 7, 10.1% of Group D)*: Group D patients without follow-up DWI. Notably, D3 had the highest median NIHSS (6) among the three subgroups; the reasons MRI was not performed in these patients (e.g., contraindications, clinical instability, or workflow factors) could not be fully reconstructed and are acknowledged as a limitation. The 90-day favorable outcome rate was 85.7%.

**Table 3:** Empirical deconstruction of Group D (imaging-quiescent) by DWI status.

Variable	D1: DWI-positive (*n* = 42)	D2: DWI-negative (*n* = 20)	D3: DWI not performed (*n* = 7)
Proportion of Group D, %	60.9	29.0	10.1
Age, years, median	60	61	62
Admission NIHSS, median (IQR)	4 (2–7)	2 (1–4)	6 (4–8)
ASPECTS, median	10	10	10
Posterior circulation involvement, *n* (%)	16 (38.1)	1 (5.0)	0 (0)
Atrial fibrillation, *n* (%)	2 (4.9)	1 (5.6)	1 (14.3)
Large-vessel occlusion, *n*/*N* (%)	2/28 (7.1)	0/12 (0)	0/5 (0)
90-day favorable outcome (mRS 0–2)	33/41 (80.5%)	16/17 (94.1%)	6/7 (85.7%)
Complete recovery (mRS = 0)	15/41 (36.6%)	11/17 (64.7%)	3/7 (42.9%)
Poor outcome (mRS ≥ 3), *n* (%)	8/41 (19.5%)	1/17 (5.9%)	1/7 (14.3%)

Proportions for posterior circulation, atrial fibrillation, and large-vessel occlusion are based on available data within each subgroup. Outcome denominators reflect availability of 90-day mRS. ASPECTS indicates Alberta Stroke Program Early CT Score; DWI, diffusion-weighted imaging; IQR, interquartile range; mRS, modified Rankin Scale; NIHSS, National Institutes of Health Stroke Scale.

### The threshold-sensitivity phenomenon

**Figure 5:**
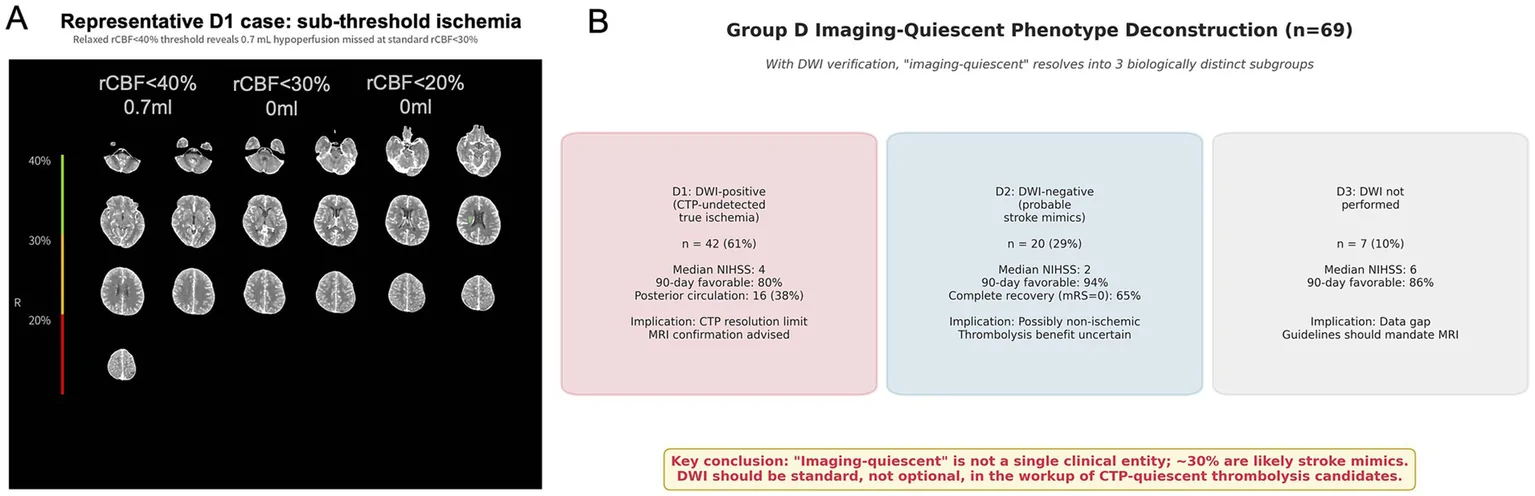
**(A)** Representative D1 case: Sub-threshold ischemia revealed by progressively relaxed rCBF cutoffs. At the standard rCBF < 30% threshold the volume is 0 mL (consistent with Group D classification); at rCBF <20% it remains 0 mL; but at rCBF< 40% a small 0.7 mL hypoperfusion focus is detected adjacent to the right lateral ventricle (small green spot, top right panel). This case had DWI-positive confirmation, demonstrating that some D1 patients harbor sub-CTP-threshold ischemia rather than truly absent ischemia. rCBF indicates relative cerebral blood flow. **(B)** Summary visualization of the three Group D subgroups (D1/D2/D3), integrating sample size, key clinical features, 90-day outcomes, and proposed clinical implications. The DWI-negative subgroup (D2) is enriched for stroke mimics and transient ischemic events, with implications for thrombolysis decision-making. DWI indicates diffusion-weighted imaging; mRS, modified Rankin Scale; NIHSS, National Institutes of Health Stroke Scale.

Although the standard rCBF<30% threshold characterizes Group D patients as having no detectable core, examination of a representative D1 case reveals a subtler phenomenon: at the more sensitive rCBF<40% threshold, a small (0.7 mL) abnormality was detected adjacent to the right lateral ventricle, which disappeared at rCBF<30% and was undetectable at rCBF<20% (Figure 5A). This sub-threshold signal corresponded to a DWI-positive ischemic lesion, suggesting that some imaging-quiescent patients may harbor early ischemia lying between operational CTP cutoffs. As this observation derives from a single illustrative case, it is presented as hypothesis-generating and would require systematic threshold-map analysis across the DWI-positive subgroup for confirmation. This finding parallels the broader debate about optimal core thresholds in mild stroke (Yedavalli et al., 2025) and suggests that protocol-level threshold adjustment, rather than DWI alone, may also identify some D1 patients prospectively.

### Whole-cohort DWI validation beyond Group D

Across all phenotypes, 24 patients were DWI-negative and 113 were DWI-positive. The DWI-negative subgroup spread across phenotypes (Group A: 2 patients, Group C: 5 patients, Group D: 20 patients), although Group D contained the substantial majority (83.3% of all DWI-negative patients), indicating that DWI-negative patients are concentrated within the imaging-quiescent phenotype. Whole-cohort comparison demonstrated meaningfully different prognostic profiles: 90-day favorable outcome was 75% in DWI-positive vs. 92% in DWI-negative patients, and complete recovery (mRS = 0) was 35% versus 52%, respectively (Figure 5B).

### Multivariable predictors of favorable outcome

In the multivariable logistic regression including NIHSS, age, sex, ASPECTS, and phenotype indicators (*n* = 161 with complete data for all model covariates and outcomes, 119 events, events-per-variable 17.0), admission NIHSS was the only consistently significant predictor of favorable 90-day outcome (OR 0.83 per point, 95% CI 0.75–0.91, *p* < 0.001; Table 4). Phenotype indicators showed direction-consistent but non-significant effects after adjustment for NIHSS, indicating that much of the between-phenotype outcome difference is captured by clinical severity. Model discrimination was good (AUC = 0.823, Pseudo *R*^2^ = 0.217).

**Table 4:** Multivariable logistic regression for 90-day favorable outcome (modified Rankin Scale 0–2). Reference group: Group A (classic mismatch).

Predictor	OR	95% CI	*p* value
Admission NIHSS (per point)	0.83	0.75–0.91	<0.001
Age (per year)	0.99	0.96–1.02	0.385
Male sex	1.33	0.53–3.33	0.548
ASPECTS (per point)	1.05	0.74–1.50	0.778
Group C vs. Group A	2.82	0.75–10.66	0.126
Group D vs. Group A	1.41	0.49–4.06	0.523

Model statistics: *n* = 161, events = 119, events-per-variable = 17, AUC = 0.823, Pseudo *R*^2^ = 0.217. ASPECTS indicates Alberta Stroke Program Early CT Score; AUC, area under the receiver operating characteristic curve; CI, confidence interval; NIHSS, National Institutes of Health Stroke Scale; OR, odds ratio.

## Discussion

### Principal findings

In a single-center thrombolysis cohort with paired CTP and DWI data, we identified three principal findings. First, the imaging-quiescent CTP phenotype is the largest single phenotype in real-world thrombolysed patients (41.1%), exceeding the classic mismatch phenotype that has historically dominated CTP-based research. Second, this imaging-quiescent phenotype is not a single clinical entity: DWI confirmation reclassifies 61% as CTP-undetected true ischemia and 29% as DWI-negative with high complete-recovery rates. Third, the DWI-positive imaging-quiescent subgroup is enriched for posterior circulation involvement, pointing to a specific clinical-imaging gap.

### Stroke mimics within thrombolysed cohorts: a quantified estimate

The prevalence of stroke mimics among thrombolysed patients in published series ranges from 1.4 to 17%, with higher rates in mild-stroke series and in centers using rapid clinical-only decision-making (Tsivgoulis et al., 2015; Liberman et al., 2015). Our finding that 29% of the imaging-quiescent phenotype—corresponding to approximately 12% of the overall thrombolysed cohort—was DWI-negative sits at the upper end of this range. The clinical profile of these patients (median NIHSS 2, 65% reaching mRS = 0 at 90 days, no documented LVO) is compatible with either non-ischemic events with full clinical resolution or transient ischemic events whose substrate had resolved before MRI. Because we did not perform independent clinical adjudication, obtain follow-up imaging, or record discharge diagnoses, this 12% figure should be read as an upper bound on the mimic fraction rather than a point estimate of it. The safety implication is direct: thrombolysis in stroke mimics confers no benefit but retains the hemorrhagic risk profile of true stroke (Tsivgoulis et al., 2015). Although our analysis cannot quantify intracranial hemorrhage risk in this cohort, the magnitude of the DWI-negative fraction we observed supports the hypothesis that post-thrombolysis DWI adds diagnostic information in CTP-quiescent patients. Because MRI in this study was obtained after thrombolysis and did not guide real-time treatment, our data cannot establish the value of pre-thrombolysis DWI, which would require prospective workflow comparison. Emerging mobile low-field MRI may eventually make DWI feasible within the pre-thrombolysis window (Xie et al., 2025), but current evidence supports technical and diagnostic feasibility rather than diagnostic equivalence with conventional MRI or clinical utility in CTP-quiescent candidates; this remains a question for prospective evaluation.

### The posterior circulation gap

Among DWI-positive imaging-quiescent patients, 16 of 42 (38.1%) had posterior circulation involvement on MR description, comprising 10 patients with unequivocal brainstem, cerebellar, or occipital lesions and 6 with thalamic lesions classified as posterior-circulation on the basis of predominant perforator supply. Standard CTP protocols, optimized for anterior circulation perfusion mapping, are well known to have reduced sensitivity in the brainstem, cerebellum, and thalamus due to skull-base artifact, smaller territorial volumes, and limited longitudinal coverage in single-acquisition protocols (Heit and Wintermark, 2016). The practical implication is that this gap causes triage workflows conditioning treatment decisions on CTP to systematically under-detect a subset of true ischemic strokes, particularly posterior-circulation events.

### The threshold-sensitivity sub-finding

Examination of representative D1 cases revealed a finer-grained phenomenon: at the more sensitive rCBF<40% threshold, small foci (~1 mL) of hypoperfusion can be detected in some patients who appear “imaging-quiescent” by standard rCBF<30% criteria (Figure 5A). This raises a practically important question for CTP protocol design: is the rCBF<30% threshold optimal in mild-stroke triage, or does the diagnostic value of CTP in this population improve with relaxed cutoffs? As this observation derives from a single illustrative case, it is presented as hypothesis-generating; systematic threshold-map analysis across the DWI-positive subgroup, and prospective comparison of threshold definitions in mild stroke, would be required to address the question properly.

### Implications for the clinical use of CT perfusion in mild stroke

Taken together, these findings invite a recalibration of how CTP information is integrated into mild-stroke decision-making, while stopping short of arguments the data cannot support. Group D is best understood not as a single forgotten entity but as a composite of CTP-undetected ischemia, resolved or non-ischemic events, and an indeterminate subgroup without confirmatory imaging, each carrying distinct prognostic implications. We observed that within a thrombolysed cohort, patients with no detectable perfusion abnormality (Group D) and patients with pure penumbra (Group C) achieved similar 90-day favorable outcomes; our formal equivalence test supports this similarity at the ±15% margin but not at the more stringent ±10% margin. This observation alone cannot establish that perfusion findings have no decisional value, particularly given the substantially different mechanical thrombectomy rates between the two groups (18.8% vs. 4.3%) and the potential for treatment effect to confound naive outcome comparisons. What our data more clearly suggest is that CTP alone cannot reliably distinguish ischemic from non-ischemic etiology in mild stroke, and that DWI provides complementary information. Any translation of this into workflow, however, requires evidence we do not have. MRI in this study was obtained after thrombolysis and did not guide real-time treatment, so our data speak to the post-thrombolysis diagnostic value of DWI and not to its pre-thrombolysis treatment-decision value. We did not compare CTP-only with CTP-plus-DWI workflows, or assess effects on treatment delay, safety, functional outcome, cost-effectiveness, or feasibility. Prospective workflow comparison is therefore required before any change to pre-thrombolysis practice can be recommended.

### Methodological implications for perfusion research

Conventional mismatch and HIR computations (Olivot et al., 2014; Guenego et al., 2019) require non-zero denominators (Figure 1). The applicability of the two ratios differs across phenotypes: in Group C (core = 0 mL, Tmax>6 s > 0 mL) the mismatch ratio is undefined while HIR remains computable, whereas in Group D (both volumes = 0 mL) both ratios are undefined. Published CTP studies commonly relegate Group C and Group D patients to “not assessable” or drop them—a missing-not-at-random pattern that biases inferences toward the LVO end of the spectrum. We therefore suggest that perfusion-based stroke research report the prevalence and outcomes of imaging-quiescent patients, and where feasible, integrate DWI confirmation to enable the kind of phenotype deconstruction reported here. We deliberately constructed the phenotype framework on the two primary thresholded volumes (rCBF<30% core and Tmax>6 s hypoperfusion) rather than on HIR, because these volumes are the direct outputs common to all CTP platforms and constitute the very denominators whose failure motivates this study; HIR is a ratio derived from those same quantities and was therefore treated not as an independent classifying dimension but as an illustration of the same mathematical limitation.

### Limitations

Several limitations require explicit acknowledgment. First, this is a single-center retrospective cohort study without a non-thrombolysed comparator group, precluding direct estimation of treatment effect within each phenotype. Second, DWI was performed on a clinical-indication basis rather than uniformly, which may have introduced selection in which Group D patients received MRI; however, the high overall DWI ascertainment rate (89.9% within Group D) limits but does not eliminate this concern. To address this directly, we compared Group D patients who did and did not undergo MRI (Supplementary Table S1). The two groups were similar in age, baseline NIHSS, ASPECTS, vascular risk factors, large-vessel occlusion, thrombectomy use, and 90-day favorable outcome (49/58, 84.5% vs. 6/7, 85.7%; *p* = 1.000). The only significant difference was a shorter door-to-needle time among patients who did not undergo MRI (median 39 vs. 46 min; *p* = 0.013), suggesting that the most rapidly treated patients were less likely to proceed to subsequent MRI rather than that MRI was withheld from a clinically distinct population. Residual confounding cannot be excluded given the small number of patients without MRI (n = 7), and D3 is best regarded as missing data rather than a distinct biological subgroup; no formal imputation-based sensitivity analysis was performed. Third, we did not capture symptomatic intracranial hemorrhage as a safety endpoint, a critical gap given that approximately 12% of thrombolysed patients in our cohort may represent stroke mimics. Fourth, several subgroup analyses—particularly Group B (*n* = 6) and D3 (*n* = 7)—involve very small numbers, so the corresponding estimates are imprecise and should be regarded as exploratory; multicollinearity among model covariates was formally assessed and was not problematic (all VIF < 2; Supplementary Table S2), although we did not perform sensitivity analyses using alternative variable-selection strategies. Fifth, two thrombolytic agents (alteplase and tenecteplase) were used during the study period (Wang et al., 2023; Menon et al., 2022); we did not stratify by agent. Sixth, posterior circulation identification was based on radiologist MR descriptions rather than systematic anatomical scoring. This approach, common in retrospective designs, may misclassify lesion territory because it depends on the completeness and wording of the original reports and does not permit standardized anatomical scoring; some posterior-circulation lesions may therefore have been over- or under-counted. Seventh, DWI lesions were characterized only at the level of the descriptive radiology report. Territorial assignment was inferred from reported anatomical structures rather than standardized vascular-territory scoring, and thalamic lesions (*n* = 6) were counted as posterior-circulation involvement on the basis of their predominant perforator supply, although thalamic vascularization is variable; restricting the definition to unequivocal brainstem, cerebellar, or occipital lesions would yield a lower estimate (10/42, 23.8%). Five patients had lesions spanning both territories. Infarct volumes were not quantified, and the reports did not consistently distinguish lacunar from cortical patterns, so these features could not be analyzed. Systematic, image-based lesion characterization is therefore a priority for future work. Finally, our cohort is from a single Chinese tertiary stroke center; generalizability requires confirmation in independent cohorts.

## Conclusion

The imaging-quiescent phenotype—patients with no measurable ischemic core or hypoperfusion on CT perfusion—comprises 41% of consecutively thrombolysed stroke patients in our cohort and is internally heterogeneous in a way that DWI can directly resolve. Approximately 61% of imaging-quiescent patients are DWI-positive, indicating CTP-undetected true ischemia disproportionately involving the posterior circulation, while approximately 29% are DWI-negative with high complete-recovery rates, a population enriched for stroke mimics or transient ischemic events but not equivalent to adjudicated mimic diagnoses. Representative CTP imaging confirms that the standard mismatch ratio formula fails mathematically in roughly 60% of cases, a fact directly observable in routine clinical software output. These findings are hypothesis-generating. Because MRI was obtained after thrombolysis and did not guide real-time treatment, our data support further prospective evaluation of DWI in CTP-quiescent thrombolysis candidates rather than an immediate change to pre-thrombolysis workflow. We suggest that stroke registries report imaging-quiescent and DWI-discordant phenotypes alongside conventional mismatch profiles.

## Data Availability

The original contributions presented in the study are included in the article/Supplementary material, further inquiries can be directed to the corresponding author.
