# Supplementary material for: The imaging-quiescent phenotype in thrombolysed acute ischemic stroke: a diffusion-weighted MRI–based deconstruction
Source: Front Neurosci. 2026 Aug 26;20:1925929. doi: 10.3389/fnins.2026.1925929 (PMC13553961; doi:10.3389/fnins.2026.1925929)
Supplement: Supplementary file 1 [file Table_1.docx]

Supplementary Table S1. Baseline characteristics of Group D (imaging-quiescent) patients with and without post-thrombolysis MRI.

| **Variable** | **MRI performed (n=62)** | **MRI not performed (n=7)** | ***P* value** | **n avail (MRI/noMRI)** |
| --- | --- | --- | --- | --- |
| Age, years | 60 (47–71) | 62 (52–68) | 0.551 | 61/7 |
| Baseline NIHSS | 4 (2–6) | 6 (4–8) | 0.107 | 62/7 |
| ASPECTS | 10 (10–10) | 10 (10–10) | 0.797 | 62/7 |
| Onset-to-treatment time, min | 173 (134–240) | 108 (84–194) | 0.136 | 59/7 |
| Door-to-needle time, min | 46 (36–60) | 39 (26–40) | 0.013 | 62/7 |
| Male sex | 38 (62.3%) | 5 (71.4%) | 1 | 61/7 |
| Hypertension | 48 (82.8%) | 6 (85.7%) | 1 | 58/7 |
| Diabetes mellitus | 16 (27.6%) | 3 (42.9%) | 0.408 | 58/7 |
| Dyslipidemia | 26 (44.8%) | 3 (42.9%) | 1 | 58/7 |
| Atrial fibrillation | 3 (5.1%) | 1 (14.3%) | 0.369 | 59/7 |
| Current smoking | 11 (18.3%) | 1 (14.3%) | 1 | 60/7 |
| Large-vessel occlusion | 2 (4.9%) | 0 (0.0%) | 1 | 41/4 |
| Mechanical thrombectomy | 2 (3.7%) | 0 (0.0%) | 1 | 54/6 |
| 90-day mRS 0–2 | 49 (84.5%) | 6 (85.7%) | 1 | 58/7 |

*Data are median (IQR) for continuous variables and n (%) for categorical variables.* *ASPECTS, Alberta Stroke Program Early CT Score; IQR, interquartile range; mRS, modified Rankin Scale; NIHSS, National Institutes of Health Stroke Scale.*
