# Supplementary material for: The imaging-quiescent phenotype in thrombolysed acute ischemic stroke: a diffusion-weighted MRI–based deconstruction
Source: Front Neurosci. 2026 Aug 26;20:1925929. doi: 10.3389/fnins.2026.1925929 (PMC13553961; doi:10.3389/fnins.2026.1925929)
Supplement: Supplementary file 2 [file Table_2.docx]

Supplementary Table S2. Multicollinearity diagnostics for the multivariable logistic regression model

| **Covariate** | **Variance inflation factor (VIF)** |
| --- | --- |
| Baseline NIHSS | 1.47 |
| Age | 1.06 |
| Male sex | 1.05 |
| ASPECTS | 1.32 |
| Phenotype B (vs A) | 1.17 |
| Phenotype C (vs A) | 1.37 |
| Phenotype D (vs A) | 1.77 |

*Model: 90-day favorable outcome (mRS 0–2) regressed on baseline NIHSS, age, sex, ASPECTS, and CTP phenotype (Group A as reference); 161 complete cases.*
